# Association of Low Birth Weight and Premature Birth With the Risk of Metabolic Syndrome: A Meta-Analysis

**DOI:** 10.3389/fped.2020.00405

**Published:** 2020-07-28

**Authors:** Lihong Liao, Youping Deng, Dongchi Zhao

**Affiliations:** Department of Pediatrics, Zhongnan Hospital of Wuhan University, Wuhan University, Wuhan, China

**Keywords:** low birth weight, meta-analysis, metabolic syndrome, premature birth, preterm

## Abstract

The association of preterm or low birth weight (LBW) with the risk of metabolic syndrome is still unclear. This study aimed to assess the association between preterm or LBW and metabolic syndrome risk according to study or participants' characteristics. PubMed, Web of Science, and EMBASE were searched for epidemiologic studies on the association published up to April 30, 2020. Pooled odds ratio (ORs) and weighted mean differences (WMDs) with 95% confidence intervals (CIs) were calculated using the random-effects model. Low birth weight was associated with an increased risk of metabolic syndrome (OR, 1.37; 95% CI, 1.17–1.61). In the subgroup analysis by study design, the pooled ORs for LBW and metabolic syndrome in the cohort and cross-sectional studies were 1.79 and 1.22. In the subgroup analysis by sex, LBW was found to be associated with an increased risk of metabolic syndrome in pooled studies including both men and women or studies including only women. The association between premature birth and risk of metabolic syndrome was significant in cohort studies (OR, 1.72; 95% CI, 1.12–2.65). Also, LBW or preterm was significantly associated with a higher Homeostasis Model Assessment of Insulin Resistance (WMD, 0.28; 95% CI, 0.19–0.36). Low birth weight and preterm might be risk factors for metabolic syndrome.

## Introduction

Metabolic syndrome is defined as a cluster of any three or more of these features: elevated waist circumference, elevated triglyceride level, reduced high-density lipoprotein cholesterol level, elevated blood pressure, elevated fasting glucose level (1–3), and insulin resistance is the pathogenesis (4, 5). The median prevalence of metabolic syndrome in the whole population was 3.3%, which ranged from 0 to 19.2% (6). Previous studies found that a cluster of symptoms of metabolic syndrome was associated with various chronic diseases, including cardiovascular disease, type 2 diabetes mellitus, and cancer at various sites (7–9). Therefore, clarifying the independent risk factors for metabolic syndrome is particularly important in the general population.

Several epidemiological studies were conducted to look for the cause of metabolic syndrome. Many dietary, behavioral, and psychological factors have been confirmed to be associated with metabolic syndrome, such as sugar-sweetened and artificially sweetened beverage intake (10), low levels of physical activity and sedentary behavior (11), and anxiety (12). In recent years, many studies found an association of several perinatal risk factors, such as low birth weight (LBW) and premature birth, with the increased risk of metabolic syndrome, but some others showed contradictory results (13–27). Therefore, this meta-analysis was conducted to evaluate the association of LBW and premature birth with metabolic syndrome. Moreover, the stratified analyses according to study design, sex, and continent were also illustrated.

## Materials and Methods

This meta-analysis was performed following the Preferred Reporting Items for Systematic Reviews and Meta-Analysis Statement Checklist (28).

### Literature Search Strategy

The databases PubMed, Web of Science, and EMBASE up to April 30, 2020, were searched, using the following terms: [(prematurity) OR (premature birth) OR (premature infant) OR (low birth weight) OR (preterm)] AND [(metabolic syndrome) OR (metabolic syndrome, components) OR (hypertension) OR (high blood pressure) OR (insulin resistance) OR (glucose intolerance) OR (obesity) OR (overweight) OR (fat mass) OR (dyslipidemia) OR (hypercholesterolemia), as text words or Medical Subject Heading terms. In addition, the reference lists of the included studies were reviewed for undetected relevant studies.

### Inclusion Criteria

The details of inclusion criteria were as follows: (1) study design: the study was original research from observational studies; (2) participants: general population; (3) exposure: LBW or premature birth; (4) comparator: normal birth weight or full-term birth; and (5) outcome: metabolic syndrome. The most recent and complete study was selected if data from the same population had been published repeatedly. The exclusion criteria were as follows: review, comments, animal experiments, patients diagnosed with other diseases, and studies that did not report the effect estimates between LBW or preterm and metabolic syndrome.

All identified studies were searched and reviewed by three investigators (L.L.H., D.Y.P., and Z.D.C.) independently. Disagreements on the eligibility of a study were resolved by consensus by the primary author (Z.D.C.) referring to the original article.

### Data Extraction and Quality Assessment

Data extraction from each study by two investigators independently included the first author's name, publication year, country where the study was conducted, study design, age, sample size, and number of cases, perinatal risk factors (LBW or premature birth), odds ratio (OR), or hazard ratio (all results were presented as OR owing to this study designed as cohort) with 95% confidence interval (CI), definition of metabolic syndrome, adjustment for potential confounding factors, cutoff value of LBW and preterm, and Homeostasis Model Assessment of Insulin Resistance (HOMA-IR). For studies that reported several multivariate adjusted ORs, the effect estimate that was maximally adjusted for potential confounders was selected. The study quality was assessed using the Newcastle–Ottawa Scale (NOS), which was based on selection (four items), comparability (one item), and outcome (three items), with a total of 0–9 stars (29).

### Statistical Analysis

The association of LBW or preterm with the risk of metabolic syndrome was assigned as categorical data, and OR with its 95% CI was calculated in an individual study before data pooling. Moreover, the potential association of LBW or preterm with HOMA-IR was assigned as a weighted mean difference (WMD) with 95% CI. All of the pooled analyses were carried out using the random-effects model because of underlying variations among included studies (30). The *I*^2^- and *P*-value for Q statistic were used to test the heterogeneity between the included studies (*I*^2^-values of 0, 25, 50, and 75% represented no, low, moderate, and high heterogeneity, respectively), and *P* < 0.10 was considered as significant heterogeneity (31). A univariate metaregression analysis was carried out to explore the potential sources of study heterogeneity (32). Subgroup analyses were performed by study design, sex, and continent where the studies were conducted, and the differences between subgroups were calculated using the interaction *P*-value, which was based on the *t*-test because of a lower number of included studies (33). An influence analysis was performed with one study removed at a time to assess the stability of the results (34). Publication bias was assessed with a visual inspection of the funnel plot and Egger test (35). The NOS was used to assess the quality of included studies (36).

All statistical analyses were performed using Stata 14.0 (StataCorp, College Station, TX, USA). All reported probabilities (*P*-values) were two-sided with a statistical significance level of 0.05.

## Results

### Literature Search and Study Characteristics

**Figure 1:**
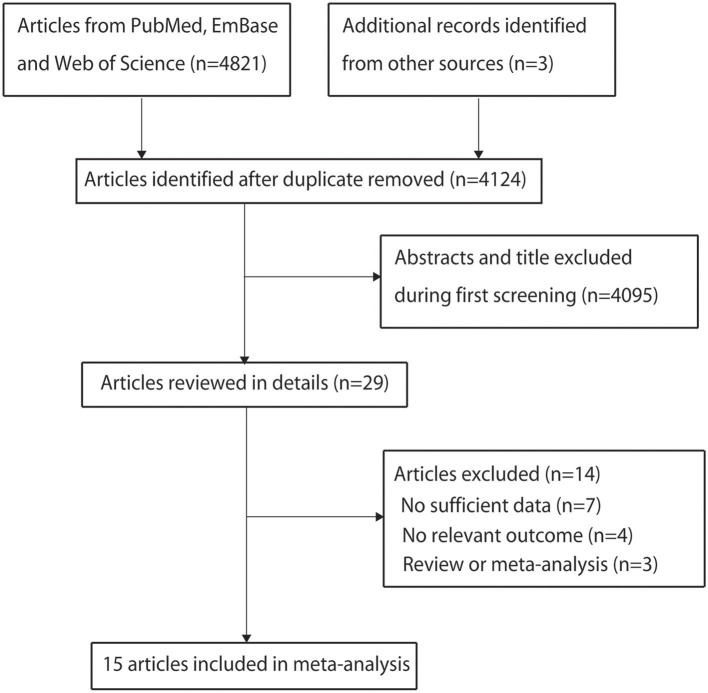
Flowchart of the selection of studies included in the meta-analysis.

The search process is shown in Figure 1. A total of 4,821 articles were identified through the literature search. Three additional articles were found from the reference lists of the included articles. A total of 700 articles were excluded owing to duplicate topics. Moreover, 4,095 articles were excluded after reviewing the titles and abstracts. After reviewing full-text articles, seven articles without OR and/or 95% CI, four articles without relevant outcome, and three reviews were excluded. Finally, 15 published articles with 16 studies were included in this meta-analysis (13–27). All but two studies (20, 27) scored 7 points, and the remaining studies scored 5 or 6 points (Table 1).

**Table 1:** Detailed characteristics of the included studies.

**References**	**Country (year)**	**Age (years)**	**Sex (male/female)**	**Study design**	**Participants (cases)**	**Perinatal risk factors**	**OR or RR (95% CI)**	**Definition of metabolic syndrome**	**Adjustment for covariates**	**Cutoff value**	**NOS**
Mi et al. (17)	China (2004)	46.5 ± 2.2	Both (494/481)	Cohort	975 (180)	LBW	OR, 1.98 (0.69–5.73)	IFG or diabetes, and presence of at least two of the following: abdominal obesity, dyslipidemia, or hypertension	Age, sex, smoking, drink, and gestational age	< P_25_	5
Wang et al. (19)	China (2016)	7–17	Both (857/913)	Cross-sectional	1,770 (19)	LBW	OR, 0.94 (0.12–7.18)	Abdominal obesity and the presence of two or more clinical features, including TAG ≥1.7 mmol/l, HDL-C <1.03 mmol/L, blood pressure ≥130/85 mm Hg, and serum FG ≥5.6 mmol/L. LBW and HBW were defined as birth weight <2,500 g and ≥4,000 g, respectively, for newborns on term without congenital malformations	Sex and age	2.5 kg	6
Ramadhani et al. (23)	Netherlands (2006)	28.4 (mean)	Both (348/396)	Cohort	722 (68)	LBW	OR, 1.80 (1.00–3.50)	Clustering of three or more of these features: WC >102 cm for men or >88 cm for women, serum triglycerides ≥150 mg/dL, serum HDL-C <40 mg/dL for men or <50 mg/dL for women, blood pressure ≥130/85 mm Hg, and serum glucose ≥110 mg/dL	Sex, family history of CVD, and participant's education	3.2 kg	6
Laaksonen et al. (22)	Finland (2003)	Exposed: 51.6 ± 6.4; unexposed: 50.4 ± 6.4	Male (462/0)	Cohort	462 (83)	LBW	OR, 2.70 (1.37– 5.34)	Insulin resistance in the top 25% of the non-diabetic population, IFG or diabetes, and presence of at least two of the following: abdominal obesity, dyslipidemia, or hypertension	Age and adult BMI	3.4 kg	6
Hirschler et al. (15)	Argentina (2008)	9.4 ± 2.1	Both (511/516)	Cross-sectional	1,027 (35)	LBW	OR, 1.06 (0.90–1.25)	Presence of ≥3 of the following five conditions: abdominal obesity; fasting triglycerides >110 mg/dL; HDL-C <40 mg/dL; blood pressure >90th percentile for age, sex, and height; fasting glucose >100 mg/dL; or use of DM medications	Age and sex	2.5 kg	5
Briskiewicz et al. (20)	Brazil (2018)	35–74	Female (0/6,872)	Cross-sectional	6,872 (NA)	LBW	OR, 1.28 (1.24–1.45)	Having at least three of the following components based on the National Cholesterol Education Program Adult Treatment Panel III updated guidelines: high waist circumference; high blood glucose; low HDL cholesterol; hypertriacylglycerolemia; and hypertension	Age, race/skin color, education, PA, smoking, alcohol consumption, relative leg length, age at menarche, and BMI at the age of 20 years	2.5 kg	6
dos Santos Alves Pde et al. (14)	Brazil (2015)	10–20	Both (64/108)	Cohort	172 (7)	LBW	OR, 0.77 (0.18–3.33)	<16.0 years: WC ≥90th percentile; high triglycerides ≥1.7 mmol/L; low HDL-C <1.03 mmol/L; blood pressure ≥130/85 mm Hg, or treatment of previously diagnosed hypertension; fasting glucose ≥5.6 mmol/L; or previously diagnosed type 2 DM; age 16–20 years: WC ≥90 cm for South American men and ≥80 cm for South American women, according to the national consensus; elevated triglycerides ≥1.7 mmol/L; reduced HDL-C, <1.03 mmol/L for men and <1.29 mmol/L for women; blood pressure ≥130/85 mm Hg, or treatment of previously diagnosed hypertension; fasting glucose ≥5.6 mmol/L; or previously diagnosed type 2 DM	Crude	2.5 kg	5
Xiao et al. (24)	China (2010)	59.3 ± 8.1	Both (990/1,029)	Cohort	2,019 (515)	LBW	OR, 1.66 (1.18–2.34)	Presence of three of the following five components: fasting glucose of at least 110 mg/dL or diagnosed DM; elevated blood pressure or history of hypertension; serum HDL-C concentration <40 mg/dL for men and <50 mg/dL for women; serum triglyceride concentration of at least 150 mg/dL; a waist circumference of at least 102 cm for men and at least 88 cm for women	Sex, age, central obesity, smoking status, alcohol intake, hypertension, dyslipidemia, family history of DM, occupational status, current social class, gestational age, and gestational hypertension	2.5 kg	6
Jornayvaz et al. (21)	Switzerland (2016)	50.2 ± 10.1	Female (0/1,458)	Cross-sectional	1,458 (210)	LBW	OR, 1.75 (1.15–2.68)	Central obesity, raised triglycerides, reduced HDL-C, raised blood pressure, raised fasting plasma glucose level	Age, smoking status, and PA	2.5 kg	6
Jornayvaz et al. (21)	Switzerland (2016)	49.7 ± 9.9	Male (1,088/0)	Cross-sectional	1,088 (276)	LBW	OR, 0.96 (0.52–1.76)	Central obesity, raised triglycerides, reduced HDL-C, raised blood pressure, raised fasting plasma glucose	Age, smoking status, and PA	2.5 kg	6
Balasuriya et al. (26)	Norway (2018)	26.4 ± 0.6 26.5 ± 0.4	Both (60/68)	Cohort	128 (12)	LBW	OR, 1.92 (0.64–5.72)	Having any three of the following: central obesity (WC ≥94 cm in men and ≥80 cm in women); triglycerides ≥1.7 mmol/L; HDL cholesterol <1.03 mmol/L in men, <1.29 mmol/L in women, or on treatment for these dyslipidemias; blood pressure ≥130/85 mm Hg, or on treatment for hypertension; fasting plasma glucose ≥5.6 mmol/L, previously diagnosed type 2 DM or on treatment for DM	Crude	1.5 kg	5
Sipola-Leppänen et al. (25)	Finland (2015)	Exposed: 23.1 ± 1.4; unexposed: 23.6 ± 1.1	Both (233/245)	Cohort	478 (27)	Premature birth	OR, 4.60 (1.90–11.10)	Three or more of the following five criteria had to be met: central obesity (WC ≥94 cm in men and ≥80 cm in women); triglycerides ≥1.7 mmol/L; HDL-C level <1.03 mmol/L in men and <1.29 mmol in women; blood pressure ≥130/85 mm Hg; and fasting plasma glucose level ≥5.6 mmol/L or type 2 DM	Sex, age, cohort, parental educational level, maternal smoking during pregnancy, birth weight standard deviation score, and parental hypertension, DM, myocardial infarction/stroke, self-reported PA, and daily smoking	34 and 37 weeks	6
Catov et al. (27)	USA (2016)	Preterm: 23 (20–26); term: 24 (21–27)	Women (0/1,205)	Cohort	1,205 (315)	Premature birth	HR: 1.41 (1.13–1.77)	Three out of the following five factors had to be met: WC > 88 cm; fasting triglycerides ≥150 mg/dL; HDL-C <50 mg/dL; blood pressure ≥130/85 mm Hg and/or on antihypertensive medication; and fasting glucose ≥100 mg/dL and/or treatment with DM medication	Blood pressure, WC, triglycerides, glucose, HDL cholesterol, age, race, education, baseline BMI, parous at baseline, smoking at baseline, time-varying parity, time-varying exposure to gestational DM or hypertensive disorders of pregnancy, and time-varying weight gain	37 weeks	7
Ramirez-Velez et al. (18)	Colombia (2017)	9–17.9	Both (1,134/1,376)	Cross-sectional	2001 (NA)	Premature birth	OR, 0.86 (0.52–1.42)	At least three of the following five criteria: TG ≥100 mg/dL; HDL-C <50 mg/dL (<45 mg/dL for boys aged 9–19 years); fasting glycemia ≥110 mg/dL; WC >75th percentile for age and sex; and systolic blood pressure 90th percentile for age, sex, and height	Age, pubertal stage, and weight status by sex	37 weeks	6
Darlow et al. (13)	New Zealand (2019)	27–29	Both (152/169)	Cohort	321 (50)	Premature birth	OR, 1.37 (0.75–2.51)	Anthropometric measurements, blood pressure, total body fat, and, following an overnight fast, standard laboratory tests for plasma glucose and free insulin, lipid screen, and hemoglobin A_1c_	Crude	28 weeks	7
Mardones et al. (16)	Chile (2014)	11.4 ± 1.0	Both (1,579/1,711)	Cohort	3,290 (239)	Premature birth	OR, 1.58 (0.68–3.68)	At least three out of five of its components were present, as defined by the following cutoff points: WC ≥90th percentile, blood pressure ≥90th percentile, low HDL-C ≤ 40 mg/dL, TG ≥110 mg/dL, and glucose ≥100 mg/dL	Percentage of fat mass, sex, and Tanner stage	37 weeks	6

*BMI, Body mass index; CI, confidence interval; CVD, cardiovascular disease; DM, diabetes mellitus; HDL-C, high-density lipoprotein cholesterol; IFG, impaired fasting glycemia; LBW, low birth weight; NA, not available; OR, odds ratio; PA, physical activity; RR, relative risk; WC, waist circumference*.

### Characteristics of Studies

For the association between LBW and the risk of metabolic syndrome, 10 articles (13–16, 18, 21–23, 25, 26) with 11 studies (six cohort studies and five cross-sectional studies) were included, involving 16,693 participants. Among these studies, three were conducted in Asia, five in Europe, and two in South America. Seven studies focused on women and men, two only on men, and two only on women. For the association between premature birth and the risk of metabolic syndrome, five articles (19, 20, 23, 24, 27) with five studies (four cohort studies and one cross-sectional study) were included, involving 7,295 participants. Among these studies, one was conducted in Europe, two in South America, one in North America, and one in Oceania. Four studies focused on women and men, and the remaining one study only on women. All of the included studies reported OR as an effect estimate, excluding the study conducted by Catov et al. (27). The detailed characteristics of the included studies are shown in Table 1.

### Quantitative Synthesis

The pooled results of the association of LBW and premature birth with the risk of metabolic syndrome are summarized in Table 2.

**Table 2:** Summary risk estimates of the association of LBW and premature with the risk of metabolic syndrome.

	**Subgroup**	**No. of studies**	**Pooled OR (95% CI)**	***I*^**2**^ (%)**	***P*_**heterogeneity**_**	***P-*value between subgroups**
LBW	**All studies**	11	1.37 (1.17–1.61)	43.6	0.060	
	**Study design**					
	Cohort study	6	1.79 (1.39–2.31)	0.0	0.712	0.007
	Cross-sectional study	5	1.22 (1.04–1.43)	46.1	0.115	
	**Continent**					
	Asia	3	1.66 (1.21–2.29)	0.0	0.817	0.023
	Europe	5	1.69 (1.23–2.33)	23.7	0.263	
	South America	3	1.18 (1.00–1.39)	55.9	0.104	
	**Gender**					
	Both	7	1.37 (1.04–1.79)	35.0	0.161	0.464
	Male	2	1.59 (0.58–4.38)	79.7	0.026	
	Female	2	1.39 (1.06–1.83)	50.7	0.154	
Premature	**All studies**	5	1.48 (1.00–2.21)	62.5	0.030	
	After excluding one study (RR >3.0)	4	1.30 (1.04–1.62)	9.5	0.345	
	**Study design**					
	Cohort studies	4	1.72 (1.12–2.65)	54.5	0.086	0.043
	Cross-sectional studies	1	0.86 (0.52–1.42)	NA	NA	

*CI, confidence interval; LBW, low birth weight; NA, not available; RR, relative risk*.

**Figure 2:**
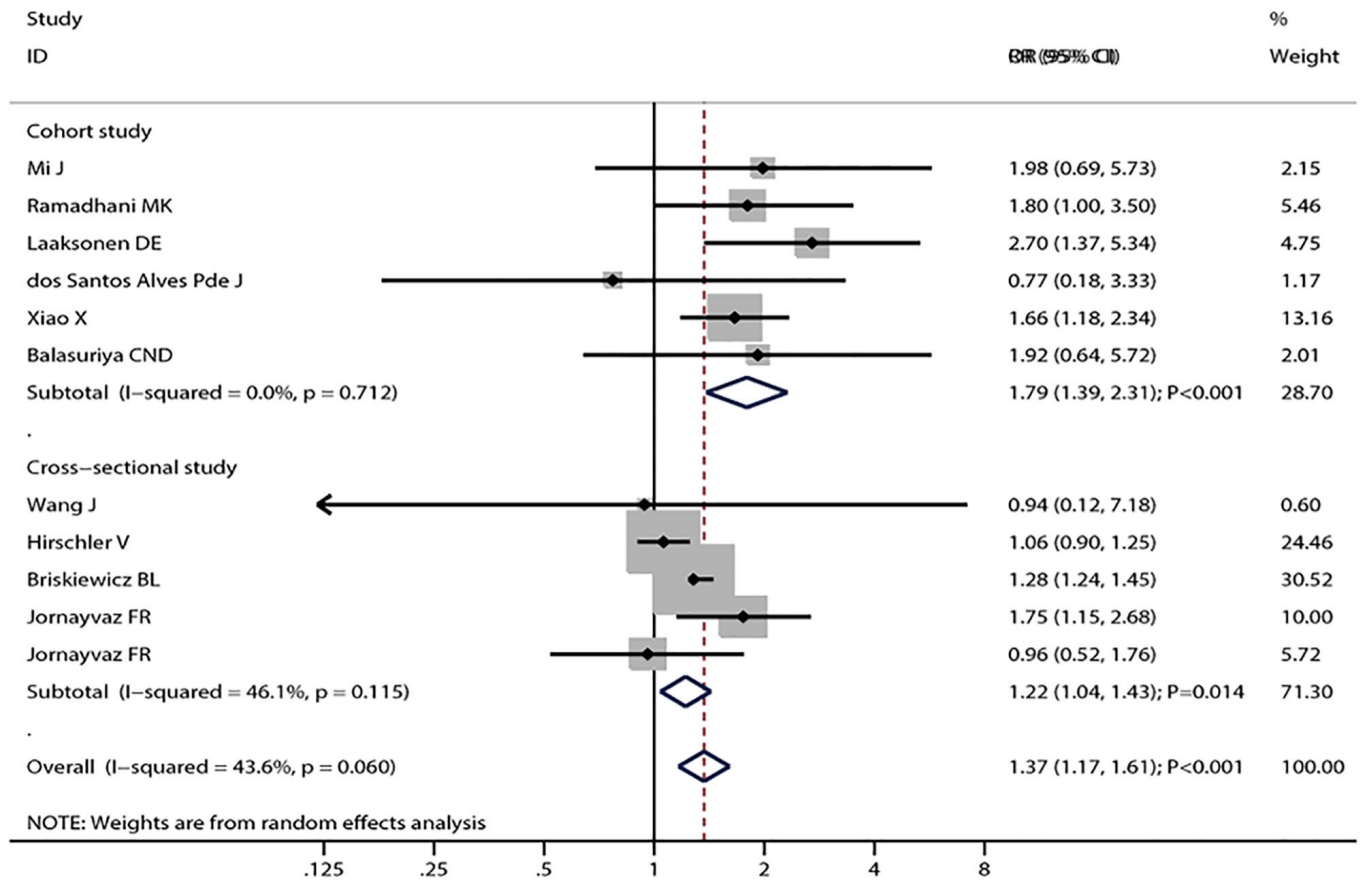
Forest plot of LBW and the risk of metabolic syndrome stratified by the study design. The size of the gray box is positively proportional to the weight assigned to each study, and horizontal lines represent 95% CIs.

**Figure 3:**
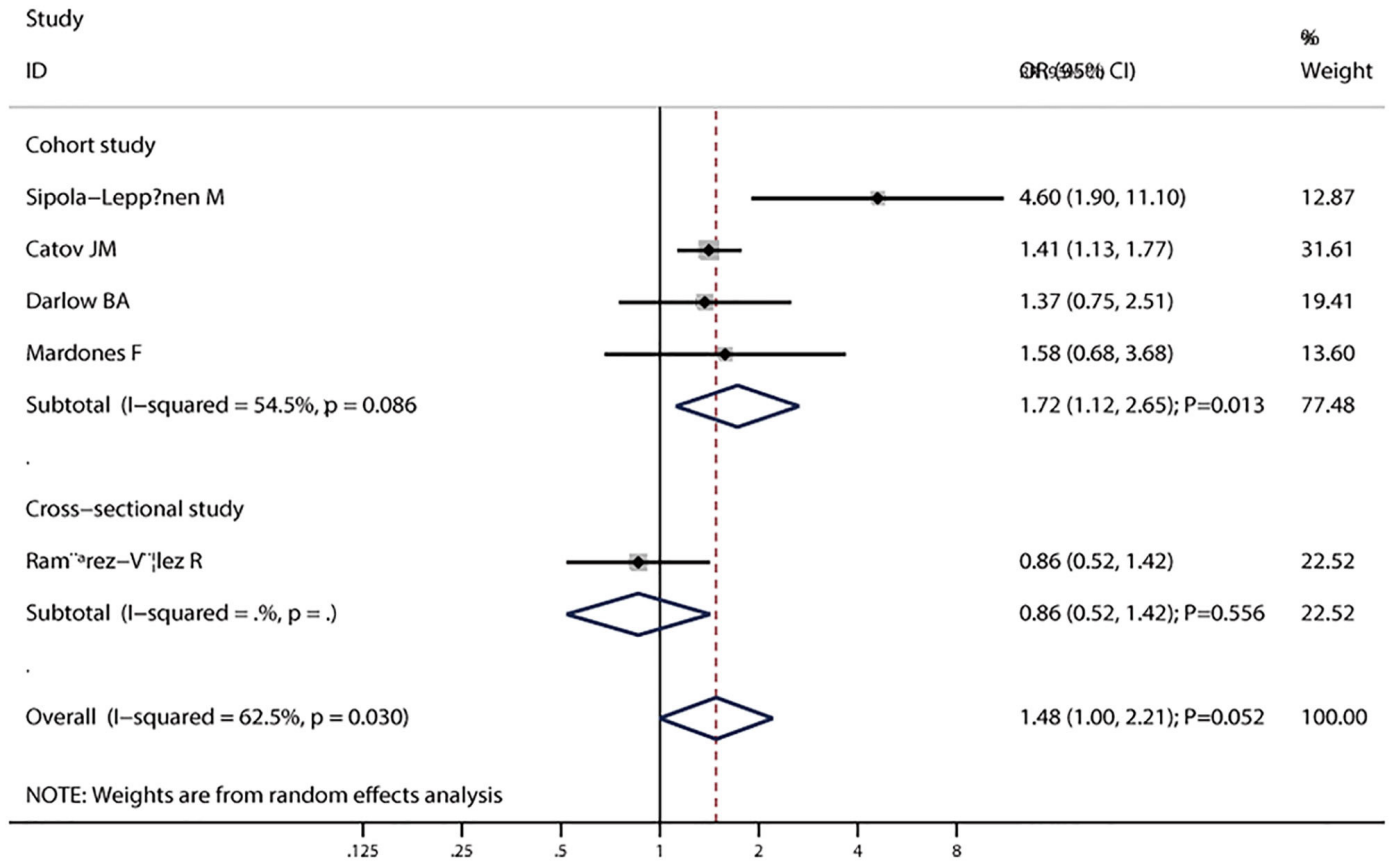
Forest plot of preterm and the risk of metabolic syndrome stratified by the study design. The size of the gray box is positively proportional to the weight assigned to each study, and the horizontal lines represent 95% CIs.

**Figure 4:**
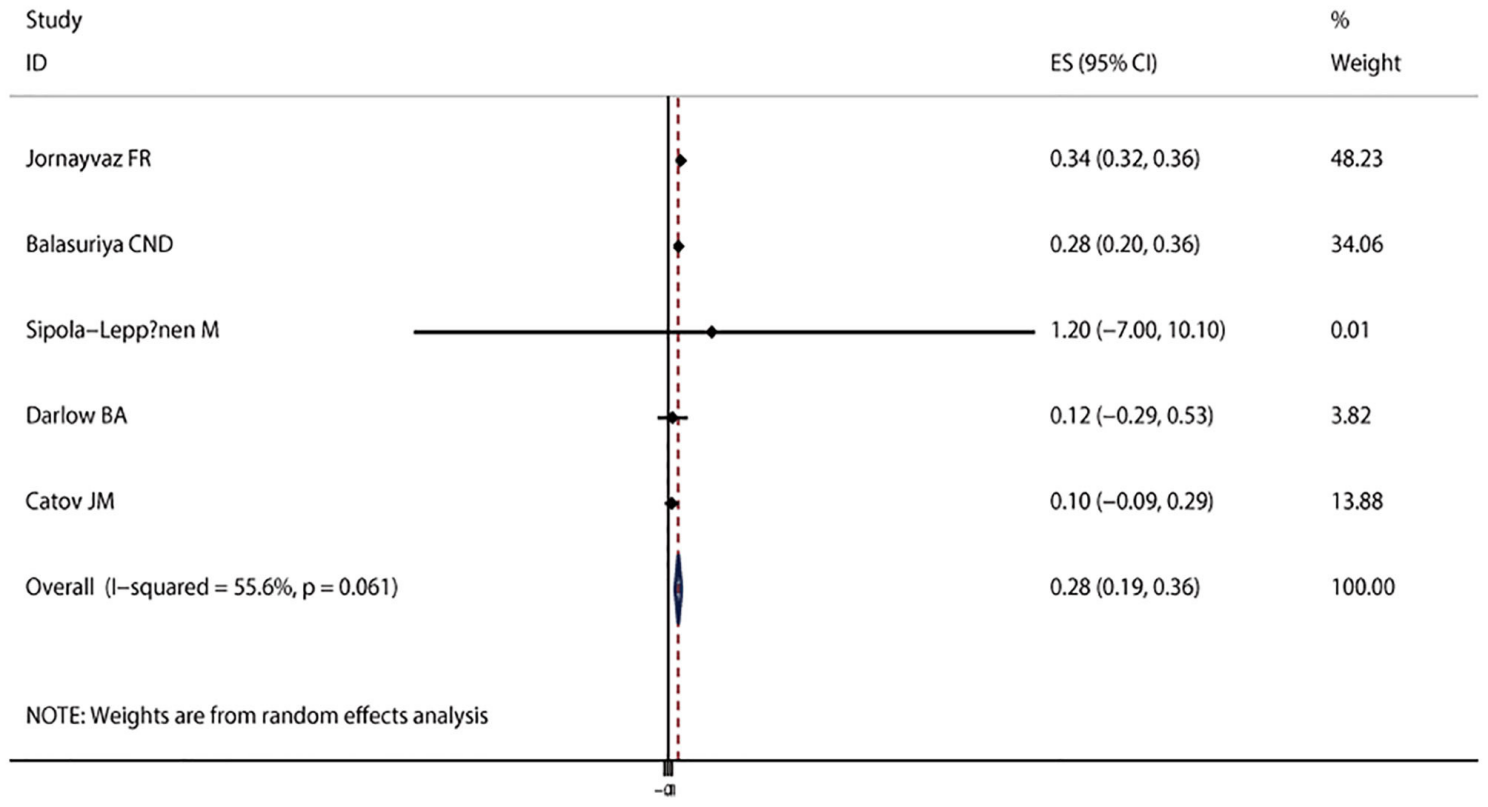
Forest plot of LBW or preterm with the insulin resistance level. The size of the gray box is positively proportional to the weight assigned to each study, and the horizontal lines represent 95% CIs.

The pooled results suggested that LBW (OR = 1.37; 95% CI, 1.17–1.61; *I*^2^ = 43.6%; *P*_heterogeneity_ = 0.060, Figure 2) was significantly associated with the risk of metabolic syndrome. In a subgroup analysis stratified by the study design, the pooled OR of cohort studies was 1.79 (95% CI, 1.39–2.31), with no evidence of heterogeneity (*I*^2^ = 0.0%; *P*_heterogeneity_ = 0.712), and the pooled OR of cross-sectional studies was 1.22 (95% CI, 1.04–1.43; *I*^2^ = 46.1%; *P*_heterogeneity_ = 0.115) (Figure 2). In the subgroup analysis stratified by continent, a significant association was found in every continent: Asia (OR = 1.66; 95% CI, 1.21–2.29; *I*^2^ = 0.0%; *P*_heterogeneity_ = 0.817), Europe (OR = 1.69; 95% CI, 1.23–2.33; *I*^2^ = 23.7%; *P*_heterogeneity_ = 0.263), and South America (OR = 1.18; 95% CI, 1.00–1.39; *I*^2^ = 55.9%; *P*_heterogeneity_ = 0.104). Significant associations were found in studies including men and women (OR = 1.37; 95% CI, 1.04–1.79; *I*^2^ = 35.0%; *P*_heterogeneity_ = 0.161), as well as women only (OR = 1.39; 95% CI, 1.06–1.83; *I*^2^ = 50.7%; *P*_heterogeneity_ = 0.154), but not in men (OR = 1.59; 95% CI, 0.58–4.38; *I*^2^ = 79.7%; *P*_heterogeneity_ = 0.026). The interaction test suggested that the study design and continent biased the association between LBW and metabolic syndrome. The pooled OR showed a non-significant positive association between premature birth and metabolic syndrome (OR = 1.60; 95% CI, 1.00–2.21; *I*^2^ = 62.5%; *P*_heterogeneity_ = 0.030, Figure 3). In the subgroup analysis stratified by the study design, the pooled OR of cohort studies was 1.72 (95% CI, 1.12–2.65), with reduced heterogeneity (*I*^2^ = 54.5%; *P*_heterogeneity_ = 0.086). Moreover, the association between preterm and metabolic syndrome differed according to the study design. Finally, the pooled WMD indicated that LBW or preterm was associated with the higher level of HOMA-IR (WMD, 0.28; 95% CI = 0.19–0.36; *I*^2^ = 55.6%; *P*_heterogeneity_ = 0.061, Figure 4).

### Metaregression and Sensitivity Analysis

To explore the sources of between-study heterogeneity, the univariate metaregression analysis was performed with the covariates of study design, sex, and continent where the study was conducted. However, none of these covariates was a potential source of between-study heterogeneity. After excluding one study in the analysis of premature birth and metabolic syndrome (OR > 3.0) (22), which included relatively younger subjects, and few individuals met the definition of metabolic syndrome, the pooled result was changed to significant association (OR = 1.30; 95% CI, 1.04–1.62; *I*^2^ = 9.5%, *P*_heterogeneity_ = 0.345).

In the influence analysis, the pooled ORs (95% CIs) of the association between LBW and the risk of metabolic syndrome ranged from 1.33 (95% CI, 1.12–1.57) to 1.48 (95% CI, 1.24–1.76). The individual study did not have an excessive influence on the pooled ORs.

### Small-Study Effect Evaluation

**Figure 5:**
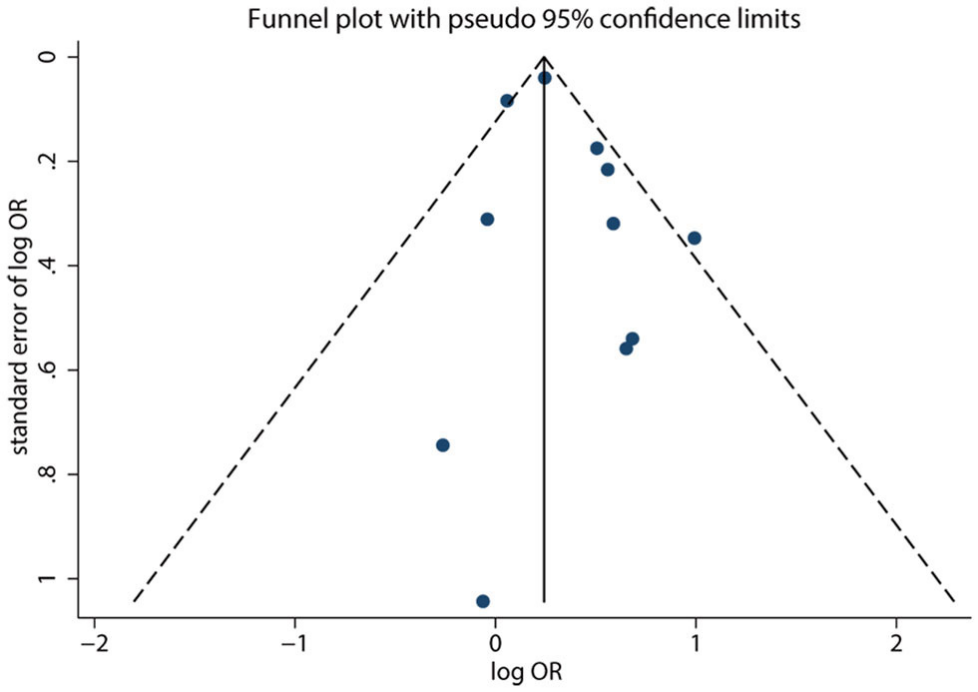
Funnel plot of LBW and the risk of metabolic syndrome. Each dot represents a different study.

The visual inspection of the funnel plot (Figure 5) and Egger test (*P* = 0.286) showed no evidence of significant small-study effect for the association between LBW and the risk of metabolic syndrome. No evidence of significant small-study effect in terms of the association between premature birth and the risk of metabolic syndrome was found (Egger test: *P* = 0.693).

## Discussion

This meta-analysis assessed the association of LBW and premature birth with the risk of metabolic syndrome. The results of the meta-analysis indicated that LBW might increase the risk of metabolic syndrome. In the subgroup analysis by study design, a significantly positive association was found in both cohort and cross-sectional studies. The pooled ORs did not indicate a statistically significant association between preterm birth and the risk of metabolic syndrome. However, the subgroup analysis by study design indicated that premature birth might be associated with an increased risk of metabolic syndrome in the cohort studies. The pooled WMD suggested a significant association between LBW or preterm and higher HOMA-IR, which is widely used in clinical and epidemiological studies to evaluate insulin sensitivity. Insulin resistance is associated with impaired glucose metabolism, increased vascular resistance, atherogenic dyslipidemia, and adipose tissue dysfunction, even before the onset of type 2 diabetes, atherosclerosis, or hypertension.

A previous meta-analysis that included 27 studies found no significant differences between preterm and term-born for the majority of outcomes associated with the metabolic syndrome, whereas preterm birth was associated with higher blood pressure in adult life (37). Moreover, Markopoulou et al. (38) conducted a meta-analysis on 43 studies and suggested that preterm birth was strongly associated with several components of metabolic syndrome and cardiovascular disease in adult life. However, these two meta-analyses investigated the associations of preterm with the components of the metabolic syndrome (38). In addition, a meta-analysis conducted by Silveira and Horta (39) found LBW was associated with an increased risk of metabolic syndrome in adults, whereas the potential impact of premature on the risk of metabolic syndrome was not illustrated. Moreover, the definition of metabolic syndrome differs owing to this study being based on earlier studies. Therefore, the present meta-analysis was conducted from another angle to evaluate the association of preterm or LBW with the risk of metabolic syndrome.

Several biological mechanisms may explain the association of LBW and premature birth with the risk of metabolic syndrome. Low birth weight and premature infants experience *in* and *ex utero* growth restriction (40). The later neonatal overfeeding may lead to rapid weight gain, which may be positively related to overweight and elevated blood glucose level (41). The third trimester of pregnancy is the critical period for the kidneys. The development of kidneys after delivery is accelerated for premature infants, and the glomeruli are morphologically abnormal, leading to the development of hypertension later in life (42). The birth weight reflects the intrauterine nutritional status to some extent. Fetal undernutrition has some effect on liver growth. Impaired liver growth may lead to permanent changes in low-density lipoprotein cholesterol metabolism (43).

Between-study heterogeneity is common and needs to be explored in meta-analyses. Moderate between-study heterogeneities were found in this meta-analysis. However, metaregression with covariates of study design, sex, and continent where the study was conducted did not find the source of between-study heterogeneities. After excluding one study (19) (OR > 3.0) in the analysis of premature birth and the risk of metabolic syndrome, the *I*^2^ declined to 9.5%, and the conclusion was changed, suggesting that the conclusion was not robust and needed further verification. Moreover, the definition of LBW and preterm differed across included studies, affecting the net effect estimates between LBW or preterm and the risk of metabolic syndrome.

The present meta-analysis had several strengths. First, it was based on a large sample size, and the findings were more robust than those of any individual study. Second, the positive associations remained when cohort studies were pooled, indicating a potential causal relationship. Third, subgroup analysis was conducted with reduced between-study heterogeneity, suggesting that the results were stable.

However, this meta-analysis also had several limitations. First, the number of studies included was insufficient, especially for the analysis of premature birth. Second, the adjusted confounders differed across included studies, which might play an important role in the risk of metabolic syndrome. Third, the information about gestational age and birth weight was obtained through self-reported questionnaires, and it differed across included studies, thus affecting the progression of metabolic syndrome. Fourth, the data on the gestational age of infants in the LBW groups were not available. Hence, whether LBW in a preterm infant had different associations than LBW in a full-term infant could not be evaluated. Fifth, the prevalence of metabolic syndrome increased with age, and the outcome assessed at various ages might have biased the results. Sixth, the definition of metabolic syndrome differed across included studies, affecting the effect estimates for the association of LBW and preterm with the risk of metabolic syndrome. Finally, the analysis was at the study level, and individual patient data were not available, which restricted more detailed analysis, including the potential interaction impacts of LBW and preterm.

In conclusion, this meta-analysis suggests that LBW might be a risk factor for metabolic syndrome in childhood and adulthood. Further high-quality studies should be conducted to assess the potential interaction impacts of LBW and preterm on the risk of metabolic syndrome.

## Data Availability Statement

The original contributions presented in the study are included in the article/supplementary material, further inquiries can be directed to the corresponding author.

## Author Contributions

LL, YD, and DZ jointly wrote the article and approved the final. All authors contributed to the article and approved the submitted version.

## Conflict of Interest

The authors declare that the research was conducted in the absence of any commercial or financial relationships that could be construed as a potential conflict of interest.
